# Levofloxacin prophylaxis in patients with newly diagnosed myeloma (TEAMM): a multicentre, double-blind, placebo-controlled, randomised, phase 3 trial

**DOI:** 10.1016/S1470-2045(19)30506-6

**Published:** 2019-12

**Authors:** Mark T Drayson, Stella Bowcock, Tim Planche, Gulnaz Iqbal, Guy Pratt, Kwee Yong, Jill Wood, Kerry Raynes, Helen Higgins, Bryony Dawkins, David Meads, Claire T Hulme, Irene Monahan, Kamaraj Karunanithi, Helen Dignum, Edward Belsham, Jeff Neilson, Beth Harrison, Anand Lokare, Gavin Campbell, Michael Hamblin, Peter Hawkey, Anna C Whittaker, Eric Low, Janet A Dunn

**Affiliations:** a School of Immunity and Infection, University of Birmingham, Birmingham, UK; b School of Sport, Exercise and Rehabilitation Sciences, University of Birmingham, Birmingham, UK; c King's College Hospital NHS Trust, London, UK; d Department of Medical Microbiology, St George's, University of London, London, UK; e Warwick Clinical Trials Unit, University of Warwick, Coventry, UK; f University Hospitals Birmingham NHS Trust, Birmingham, UK; g Department of Haematology, UCL Cancer Institute, London, UK; h Academic Unit of Health Economics, University of Leeds, Leeds, UK; i University Hospitals North Midlands NHS Trust, Stoke On Trent, UK; j Portsmouth Hospitals NHS Trust, Portsmouth, UK; k The Dudley Group NHS Foundation Trust, Russells Hall Hospital, Dudley, UK; l University Hospitals Coventry and Warwickshire, Coventry, UK; m East Suffolk and North Essex NHS Foundation Trust, Colchester, UK; n West Midlands Public Health Laboratory, Heart of England NHS Trust, Birmingham, UK; o Patient Advocacy, Myeloma UK, Edinburgh UK

## Abstract

**Background:**

Myeloma causes profound immunodeficiency and recurrent, serious infections. Around 5500 new cases of myeloma are diagnosed per year in the UK, and a quarter of patients will have a serious infection within 3 months of diagnosis. We aimed to assess whether patients newly diagnosed with myeloma benefit from antibiotic prophylaxis to prevent infection, and to investigate the effect on antibiotic-resistant organism carriage and health care-associated infections in patients with newly diagnosed myeloma.

**Methods:**

TEAMM was a prospective, multicentre, double-blind, placebo-controlled randomised trial in patients aged 21 years and older with newly diagnosed myeloma in 93 UK hospitals. All enrolled patients were within 14 days of starting active myeloma treatment. We randomly assigned patients (1:1) to levofloxacin or placebo with a computerised minimisation algorithm. Allocation was stratified by centre, estimated glomerular filtration rate, and intention to proceed to high-dose chemotherapy with autologous stem cell transplantation. All investigators, patients, laboratory, and trial co-ordination staff were masked to the treatment allocation. Patients were given 500 mg of levofloxacin (two 250 mg tablets), orally once daily for 12 weeks, or placebo tablets (two tablets, orally once daily for 12 weeks), with dose reduction according to estimated glomerular filtration rate every 4 weeks. Follow-up visits occurred every 4 weeks up to week 16, and at 1 year. The primary outcome was time to first febrile episode or death from all causes within the first 12 weeks of trial treatment. All randomised patients were included in an intention-to-treat analysis of the primary endpoint. This study is registered with the ISRCTN registry, number ISRCTN51731976, and the EU Clinical Trials Register, number 2011-000366-35.

**Findings:**

Between Aug 15, 2012, and April 29, 2016, we enrolled and randomly assigned 977 patients to receive levofloxacin prophylaxis (489 patients) or placebo (488 patients). Median follow-up was 12 months (IQR 8–13). 95 (19%) first febrile episodes or deaths occurred in 489 patients in the levofloxacin group versus 134 (27%) in 488 patients in the placebo group (hazard ratio 0·66, 95% CI 0·51–0·86; p=0·0018. 597 serious adverse events were reported up to 16 weeks from the start of trial treatment (308 [52%] of which were in the levofloxacin group and 289 [48%] of which were in the placebo group). Serious adverse events were similar between the two groups except for five episodes (1%) of mostly reversible tendonitis in the levofloxacin group.

**Interpretation:**

Addition of prophylactic levofloxacin to active myeloma treatment during the first 12 weeks of therapy significantly reduced febrile episodes and deaths compared with placebo without increasing health care-associated infections. These results suggest that prophylactic levofloxacin could be used for patients with newly diagnosed myeloma undergoing anti-myeloma therapy.

**Funding:**

UK National Institute for Health Research.

## Introduction

Myeloma is a cancer of bone marrow plasma cells that causes anaemia, skeletal damage, renal impairment, and profound immunodeficiency,^1^ and the median age at diagnosis is 70 years.^2^, ^3^ Myeloma accounts for 2% of all cancers in the UK.^4^ Substantial advances in anti-myeloma therapy have improved survival to 50% at 5 years; however, infection contributes to death in a fifth of patients with myeloma.^1^, ^2^, ^5^, ^6^ The risk of infection is greatest in the first 3 months after diagnosis, with a third of patients suffering serious bacterial infection, and infection contributing to half of early mortality.^7^, ^8^, ^9^ Despite some reduction in early deaths with use of novel anti-myeloma agents,^5^ early mortality remains a problem and population data for England in 2011–15 showed that 5% of 22 504 patients newly diagnosed with myeloma died within the first month of diagnosis, and 21% within the first 12 months.^9^ Antimicrobial prophylaxis might reduce death from infections, since it has been shown to improve survival in patients with prolonged neutropenia.^10^ However, concerns about increased antibiotic resistance,^11^, ^12^ drug-related side-effects, and the risk of health care-associated infections^13^ mean the use of quinolone prophylaxis remains controversial.^14^, ^15^, ^16^, ^17^

**Research in context**

**Evidence before this study**

We searched PubMed for research articles published in English with the search terms “cancer”, “myeloma”, “multiple myeloma”, “early mortality, “infection”, “antibiotic prophylaxis”, “quinolones”, and “levofloxacin”. No date restrictions were applied. We found that the risk of infection was greatest in the first 3 months after diagnosis of myeloma, with a third of patients suffering serious bacterial infection and infection, contributing to half of early mortality. Antimicrobial prophylaxis, especially with quinolones, is effective at reducing infections and mortality in patients with neutropenia. However, concerns about increased antibiotic resistance and the risk of health care-associated infections linked to antibiotic prophylaxis means its application remains controversial. Despite this increasing concern about health care-associated infections, to our knowledge, there have been only two small inconclusive randomised controlled trials of antimicrobial prophylaxis in patients with myeloma, meaning a study of antimicrobial prophylaxis in patients with newly diagnosed myeloma was warranted.

**Added value of this study**

To our knowledge, this is the first double-blind randomised placebo-controlled trial of antibiotic prophylaxis in patients with myeloma and the first trial to show improved survival and reduced infections. The trial was designed to map onto current standard practice in the UK, recruiting 977 patients from 93 hospitals and allowing a choice of anti-myeloma therapy, with most patients receiving newer anti-myeloma therapies. Examination of stool samples and nasal swabs every 4 weeks revealed no difference between the levofloxacin group and the placebo group for new acquisitions of *Clostridium difficile*, meticillin-resistant *Staphylococcus aureus*, and extended-spectrum β-lactamase-positive Gram-negative rods when assessed up to 16 weeks. The results of this study suggest that the benefits of levofloxacin prophylaxis outweigh the perceived risks in patients with newly diagnosed myeloma.

**Implications of all the available evidence**

To our knowledge, this is the largest trial to date of levofloxacin prophylaxis in patients newly diagnosed with myeloma and provides the best available evidence to suggest that levofloxacin prophylaxis could be a standard of care in the first 12 weeks after diagnosis.

Although the profile of bacterial infections is similar to that seen in neutropenia, most infections in patients with myeloma are not associated with low neutrophil counts but arise because of immunodeficiency, encompassing reduced integrity of barriers and reduced competence of both innate and adaptive immunity. Given this difference and there being, to our knowledge, only two small inconclusive randomised controlled trials of antimicrobial prophylaxis in myeloma so far,^18^, ^19^ and considering the increasing concern of health care-associated infections, there was ambivalence for the use of antimicrobial prophylaxis in patients newly diagnosed with myeloma in the UK.^18^, ^19^ Here, we report the results of the tackling early morbidity and mortality in myeloma (TEAMM) trial, which assessed the benefit of 12 weeks of levofloxacin prophylaxis and the effect on resistant organism carriage and health care-associated infections in patients with newly diagnosed myeloma.

## Methods

### Study design and participants

TEAMM was a multicentre, double-blind, placebo-controlled, randomised, phase 3 trial done in 93 UK hospitals (appendix pp 1–4). Eligible patients were aged 21 years and older with newly diagnosed, symptomatic myeloma based on internationally agreed criteria,^20^, ^21^ within 14 days of starting a programme of anti-myeloma therapy. All performance statuses were permitted with no indication of life expectancy specified. Patients with the following characteristics were ineligible for this trial: contraindication to levofloxacin (ie, known to have sensitivity or allergy to levofloxacin or other quinolones, a history of tendon disorders related to fluoroquinolone administration, receiving other prophylactic antibiotic treatment [excluding pneumocystis prophylaxis if regarded as essential], receiving amiodarone or arsenic trioxide, and on active antiepileptic treatment); women of childbearing age who were not willing to use appropriate methods of contraception to prevent pregnancy; women who were breastfeeding; patients thought to have mandatory requirement for prophylactic antibiotics (with the exception of pneumocystis prophylaxis if regarded as essential); and previous (<5 years since diagnosis) or concurrent active malignancies, except surgically removed basal or squamous cell carcinoma of the skin, treated carcinoma in-situ of the breast or cervix, or incidental histological finding of prostate cancer (TNM stage T1a or 1b). Patients with remote histories (>5 years) of other cured (on no active treatment for the previous malignancy) malignancies were eligible. Patients were recruited by clinicians and research teams at their local hospitals.

The study was approved by the UK Coventry and Warwickshire Multi-Research Ethics Committee on July 29, 2011. All patients provided written, informed consent.

### Randomisation and masking

We randomly assigned patients (1:1) to levofloxacin or placebo with a computerised minimisation algorithm that generated a trial number and drug pack allocation for each patient. Allocation was stratified by centre, estimated glomerular filtration rate (>50 mL/min, 20–50 mL/min, and <20 mL/min), and intention to proceed to high-dose chemotherapy with autologous stem cell transplantation (yes *vs* no). The trial statistician allocated the term active or placebo in a 50:50 split to a list of 1500 randomly generated drug pack numbers. The password-protected list was sent to the drug packaging company who put the active drugs or placebo in correctly labelled packs. This list was then used to build the bespoke randomisation and drug inventory system. Participants were enrolled by research staff at each centre who used a dedicated randomisation phone line at the clinical trials unit and after undergoing eligibility checks were allocated a drug pack number. All investigators, patients, laboratory, and trial co-ordination staff were masked to the treatment allocation. Tablets were manufactured specifically for the trial and packaged in identical blister packs by Modepharma (Beckenham, UK). Sample drug packs were tested independently to confirm the active drug and placebo were correctly coded.

### Procedures

Symptomatic myeloma was diagnosed according to British Committee for Standards in Haematology and UK Myeloma Forum Guidelines on the Management and Diagnosis of Multiple Myeloma^20^, ^21^ with immunofixation of serum and urine to identify monoclonal immunoglobulin, measurement of bone marrow plasma cells, and identification of myeloma-related end organ damage.

Patients were given 500 mg of levofloxacin (two 250 mg tablets), orally once daily for 12 weeks, or placebo tablets (two tablets, orally once daily for 12 weeks), with dose reduction according to estimated glomerular filtration rate every 4 weeks. Adherence to trial treatment was monitored with patient diary cards. Other prophylactic antibiotics were prohibited, except for the use of low-dose co-trimoxazole to prevent pneumocystis pneumonia according to local practice. Anti-myeloma therapy was given according to local practice.

We assessed nasal swabs and stool samples collected at baseline, 4 weeks, 8 weeks, 12 weeks, and 16 weeks as per protocol for carriage of meticillin-resistant *Staphylococcus aureus* (MRSA), *Clostridium difficile*, and extended-spectrum β-lactamase (ESBL) Gram-negative coliforms. We identified toxigenic strains of *C difficile* by culture and ribotyping. We identified ESBL Gram-negative coliforms from faecal screens and clinical specimens by culture on selective agar and genotyped for CTX-M β-lactamase genes by denaturing high-performance liquid chromatography. We cultured nasal swabs for MRSA and isolates were typed and stored.

We assessed myeloma activity and markers of immunocompetence in blood and urine collected at baseline, 4 weeks, 8 weeks, 12 weeks, 16 weeks, and 1 year. Markers of immunocompetence assessed were monoclonal and polyclonal immunoglobulins, antibodies specific to a range of bacterial and viral target antigens, markers of inflammation, and cytokines.

Capture of febrile episodes was via hospital records or patient diary cards as patients were asked to self-report temperature daily or when they felt unwell. All hospitalisations were cross-referenced, and notes were checked for any additional information. Patients were treated according to local practice. Management of neutropenic sepsis is relatively standardised across the UK according to National Institute for Health and Care Excellence guidelines.^22^

Deaths were reviewed by a masked independent clinical reviewer not linked to the trial. Cause of death was ascertained from information sent by the centre regarding cause of death and disease status, and review of previous serious adverse events and myeloma response on blood samples received. Assessments were done by two trial clinicians who were masked to the trial intervention.

### Outcomes

The primary outcome was time to first febrile episode or death from all causes within the first 12 weeks of trial treatment. A febrile episode was defined as a single oral temperature of 38°C or higher that caused the patient to be given anti-infectives. A single febrile episode was defined as the initial febrile event and any subsequent fevers until that course of anti-infectives was stopped.

Secondary outcomes assessed from start of trial treatment to 12 weeks were the number of deaths and infection-related deaths; number of non-febrile infections (clinically suspected infections without a temperature ≥38°C or higher and where anti-infectives were prescribed); number of days in hospital; number of days in hospital on anti-infective; carriage and invasive infections with MRSA, *C difficile*, and ESBL Gram-negative coliforms; patient characteristics, steroid usage, and indices of immunocompetence and their relation to colonisation by and development of infection from *S aureus, C difficile*, and ESBL Gram-negative coliforms and non-health care-associated infection and Eastern Cooperative Oncology Group performance status; number of clinically documented total infections, episodes of severe sepsis (Common Terminology Criteria for Adverse Events grade 3 or 4), and suspected infections; incidence of microbiologically proven infections, the pathogens, and their susceptibility to antibiotics; days on antibiotic therapy for treatment of infection; and response to anti-myeloma therapy and its relationship to infection. Secondary outcomes from randomisation to beyond 12 weeks were carriage and invasive infections with *S aureus, C difficile*, and ESBL Gram-negative coliforms between 12 weeks and 16 weeks to assess for delayed effects from the intervention that was stopped at 12 weeks; response to anti-myeloma therapy at 16 weeks; quality of life (4-weekly questionnaires up to 16 weeks); health economics (daily diary card that captures elements of health resource use combined with information captured on the case report form); and overall survival. Number of days in hospital, days on antibiotic therapy for treatment of infection, reponse to anti-myeloma therapy at 16 weeks, health economics and quality of life, more detailed microbiology, and in-depth analysis on patient measures of immunocompetence and relation to infection, and depth of myeloma response in relation to antibiotic use, will be reported separately.

### Statistical analysis

Our power calculations assumed that 30% of patients would have a febrile episode or death in the first 12 weeks and levofloxacin would reduce this to 20%; thus, 800 patients (400 in each study group) would allow differences in excess of 10% to be detected with 90% power using a two-sided test at 5% significance. A pre-planned early stopping guideline was applied for safety and reviewed by the data and safety monitoring committee, who endorsed the continuation of enrolment from 800 patients to up to 1000 patients. Recruiting 1000 patients into the trial (500 in each group) would allow differences greater than 8% to be detected with 90% power using a two-sided test at 5% significance. 1000 patients would also allow detection of a levofloxacin-induced three-times increase in *C difficile*-positive stools from 5% to 15% between entry to the trial and 12 weeks, with 95% power and 5% significance (two-sided test).

We analysed the primary endpoint with a log-rank comparison, beginning from the date the patient started trial treatment to the time of an event, or to censor date for those with no events up to 12 weeks. All randomly assigned patients were included in an intention-to-treat analysis of the primary endpoint, assessed using Kaplan-Meier curves and the log-rank test.^23^ We did prespecified exploratory analyses with Cox proportional hazards models to compare trial groups after adjustment for stratification variables with assumptions for non-proportionality.^24^ Proportionality assumptions were checked by visual inspection of Kaplan-Meier curves. We generated forest plots to examine the treatment effects in prespecified subgroups.

We calculated overall survival from the date the patient started trial treatment to the date of death or censoring, as appropriate, with all-cause mortality. We assessed health care-associated infections with χ^2^ tests with continuity adjustments.

Statistical analyses were done with SAS (version 9.4). This trial is registered with the ISRCTN registry, number ISRCTN51731976, and the EU Clinical Trials Register, number 2011-00366-35.

### Role of the funding source

The funder and sponsors of the study had no role in study design, data collection, data analyses, data interpretation, or writing of the report. The corresponding author had full access to the data in the study and with SB, TP, GI, and JAD had final responsibility for the decision to submit for publication, with the agreement of all other authors and the data monitoring and safety committee.

## Results

**Figure 1:**
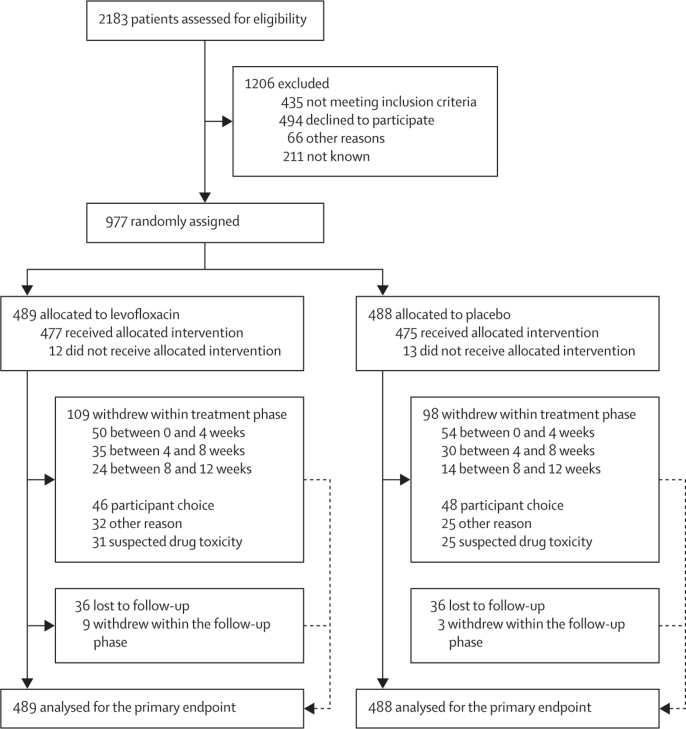
Trial profile

Between Aug 15, 2012, and April 29, 2016, we randomly assigned 977 patients to receive levofloxacin prophylaxis (489 patients) or placebo (488 patients; figure 1; appendix pp 1–4). 219 (23%) of 977 patients withdrew during the study. Because 207 (21%) of 977 patients withdrew during the trial treatment period and recruitment was faster than expected, the recruitment target was extended to 1000 patients (figure 1). Patient characteristics were similar across treatment groups in terms of stratification variables, prognostic factors, and anti-myeloma therapy, with 98% receiving immunomodulatory imide drugs or proteasome inhibitors (table 1). The median patient age was 67 years (IQR 59–75) in the levofloxacin group and 67 years (61–75) in the placebo group.

**Table 1:** Patient characteristics

		**Levofloxacin group (n=489)**	**Placebo group (n=488)**
Age (years)		67 (59–75)	67 (61–75)
Sex			
	Male	316 (65%)	295 (60%)
	Female	173 (35%)	193 (40%)
Ethnicity			
	White	452 (92%)	437 (90%)
	Mixed	1 (<1%)	2 (<1%)
	Asian or British Asian	10 (2%)	17 (3%)
	Black or Black British	26 (5%)	28 (6%)
	Chinese or other	0	1 (<1%)
	Missing	0	3 (1%)
Performance status at randomisation			
	0	164 (34%)	173 (35%)
	1	209 (43%)	188 (39%)
	2	80 (16%)	76 (16%)
	3	24 (5%)	36 (7%)
	4	2 (<1%)	5 (1%)
	Missing	10 (2%)	10 (2%)
International Staging System			
	Stage I	100 (20%)	116 (24%)
	Stage II	188 (38%)	165 (34%)
	Stage III	121 (25%)	130 (27%)
	Missing	80 (16%)	77 (16%)
Estimated glomerular filtration rate (mL/min)			
	>50	369 (75%)	369 (76%)
	20–50	95 (19%)	93 (19%)
	<20	25 (5%)	26 (5%)
High-dose CT with planned autologous stem cell transplantation			
	No	223 (46%)	222 (45%)
	Yes	266 (54%)	266 (55%)
Previous infection			
	*Clostridium difficile*	2 (<1%)	1 (<1%)
	Meticillin-resistant *Staphylococcus aureus*	6 (1%)	7 (1%)
	Extended-spectrum β-lactamase coliforms	3 (1%)	5 (1%)
Anti-infectives in previous month			
	No	332 (68%)	331 (68%)
	Yes	75 (15%)	76 (16%)
	Missing	82 (17%)	81 (17%)
Steroids 14 days before randomisation			
	Yes	248 (51%)	246 (50%)
Corticosteroids			
	Prednisolone	24 (5%)	18 (4%)
	Dexamethasone	226 (46%)	229 (47%)
	Other	..	2 (<1%)
Planned anti-myeloma treatment			
	Thalidomide-based	208 (43%)	216 (44%)
	Bortezomib-based	152 (31%)	150 (31%)
	Lenalidomide-based	72 (15%)	69 (14%)
	Lenalidomide and carfilzomib-based	50 (10%)	43 (9%)
	Other	7 (1%)	10 (2%)
Bisphosphonate status at randomisation			
	Not given	68 (14%)	60 (12%)
	Given or will be given	419 (86%)	419 (86%)
	Missing	2 (<1%)	9 (2%)
Bisphosphonate			
	Zolendronate	284 (68%)	280 (67%)
	Pamidronate	111 (26%)	105 (25%)
	Clodronate	14 (3%)	23 (5%)
	Other	3 (1%)	7 (2%)
	Missing	7 (2%)	4 (1%)
Prophylactic antiviral or antifungal			
	No	170 (35%)	166 (34%)
	Yes	237 (48%)	240 (49%)
	Missing	82 (17%)	82 (17%)
Corrected calcium (μmol/L)			
	<2·50	340 (69%)	354 (73%)
	2·50–2·75	102 (21%)	95 (19%)
	>2·75	29 (6%)	25 (5%)
	Missing	18 (4%)	14 (3%)
Evidence of bone disease			
	Yes	338 (69%)	351 (72%)
Site of bone disease			
	Vertebral fracture or collapse	118 (24%)	144 (30%)
	Lytic lesions	234 (48%)	246 (50%)
	Fractured rib	33 (7%)	24 (5%)
	Osteoporosis	38 (8%)	37 (8%)
	Other fracture	43 (9%)	41 (8%)
Serum β-2-microglobulin (mg/L)			
	≤4	189 (39%)	192 (39%)
	4–8	148 (30%)	152 (31%)
	>8	73 (15%)	67 (14%)
	Missing	79 (16%)	77 (16%)
Haemoglobin (g/dL)			
	<7·5	9 (2%)	13 (3%)
	7·5–10·0	163 (33%)	166 (34%)
	>10·0	315 (64%)	306 (63%)
	Missing	2 (<1%)	3 (1%)
Platelets (× 10^9^/L)			
	≤150	69 (14%)	79 (16%)
	>150	418 (85%)	405 (83%)
	Missing	2 (<1%)	4 (1%)
Neutrophils (× 10^9^/L)			
	<1·8	35 (7%)	55 (11%)
	1·8–3·0	138 (28%)	133 (27%)
	>3·0	312 (64%)	296 (61%)
	Missing	4 (1%)	4 (1%)
Lymphocytes (×10^9^/L)			
	<1·2	143 (29%)	147 (30%)
	1·2–1·8	165 (34%)	170 (35%)
	>1·8	177 (36%)	167 (34%)
	Missing	4 (1%)	4 (1%)
Serum creatinine (μmol/L)			
	<130	387 (79%)	384 (79%)
	130–199	57 (12%)	54 (11%)
	>199	42 (9%)	43 (9%)
	Missing	3 (1%)	7 (1%)

Data are median (IQR) or n (%).

31 (6%) of 489 patients in the levofloxacin group and 25 (5%) of 488 patients in the placebo group withdrew because of suspected drug toxicity, and 15 (2%) of these were unblinded. 24 (2%) of 977 total patients took an incorrect dose of levofloxacin (or placebo) during a 4-week block of the 12-week trial period according to their estimated glomerular filtration rate results. 40 (4%) of 977 patients required a dose reduction—23 (5%) of 489 patients in the levofloxacin group and 17 (3%) of 488 patients in the placebo group because of a reduction in estimated glomerular filtration.

597 serious adverse events were reported up to 16 weeks from the start of trial treatment (308 [52%] of 597 total events for the 489 patients in the levofloxacin group *vs* 289 [48%] of 597 total events for the 488 patients in the placebo group), with 537 (90%) reported as unlikely to be related or unrelated to study drug (144 unrelated to study drug and 126 unlikely to be related to study drug in the levofloxacin group; 147 unrelated to study drug and 120 unlikely to be related to study drug in the placebo group), and severity similar between the trial groups (appendix p 5). 29 (9%) of 308 serious adverse events in the levofloxacin group and 15 (5%) of 289 serious adverse events in the placebo group were classed as possibly related to drug, eight (3%) of 308 serious adverse events in the levofloxacin group and six (2%) of 289 serious adverse events in the placebo group were classed as probably related to drug, and one (<1%) case of delirium definitely related to drug was reported in the levofloxacin group. All 105 symptoms associated with these 59 serious adverse events that were at least possibly related to trial drugs are shown in table 2. Tendonitis was reported in five (1%) of 489 patients in the levofloxacin group (*vs* none in the placebo group) and all episodes resolved, although three had sequelae reported in the short term. One (<1%) case of suspected levofloxacin-induced confusion reversed on stopping the drug. All serious adverse events were rereviewed independently by two masked investigators after alerts from The European Medicines Agency's Pharmacovigilance Risk Assessment Committee (2018) and the US Food and Drug Administration body (2018) and no further signal was detected. Other categories of serious adverse events were similar between the study groups (table 2).

**Table 2:** Treatment-related serious adverse events

	**Levofloxacin group (n=489)**			**Placebo group (n=488)**		
	Grade 1–2 (n=37)	Grade 3 (n=26)	Grade 4 (n=8)	Grade 1–2 (n=17)	Grade 3 (n=14)	Grade 4 (n=3)
**Blood and lymphatic system disorders**						
Anaemia	2 (5%)	2 (8%)	0	0	0	0
Febrile neutropenia	0	0	1 (13%)	0	0	0
**Cardiac disorders**						
Atrial fibrillation	0	1 (4%)	1 (13%)	0	0	0
Sinus bradycardia	1 (3%)	0	0	0	1 (7%)	0
**Ear and labyrinth disorders**						
Vertigo	0	0	0	1 (6%)	0	0
**Gastrointestinal disorders**						
Abdominal distension	0	1 (4%)	0	0	0	0
Abdominal pain	0	0	0	1 (6%)	0	0
Diarrhoea	3 (8%)	2 (8%)	0	3 (18%)	5 (36%)	0
Nausea	3 (8%)	1 (4%)	0	0	0	0
Gastric obstruction	1 (3%)	0	0	0	0	0
Vomiting	3 (8%)	1 (4%)	0	0	1 (7%)	0
Other	0	0	0	1 (6%)	0	0
**General disorders and administration site conditions**						
Chills	1 (3%)	0	0	0	0	0
Fever	5 (14%)	0	0	3 (18%)	2 (14%)	0
**Immune system disorders**						
Allergic reaction	1 (3%)	0	0	0	1 (7%)	0
**Infections and infestations**						
Bladder infection	0	1 (4)	0	0	0	0
Infectious enterocolitis	1 (3%)	0	0	0	0	0
Lung infection	0	2 (8%)	0	0	0	0
Pustular rash	0	0	0	1 (6%)	0	0
Sepsis	0	0	2 (25%)	0	0	1 (33%)
Small intestine infection	0	1 (4%)	0	0	0	0
**Injury, poisoning, and procedural complications**						
Vascular access complication	0	1 (4%)	0	0	0	0
**Investigations**						
Increased alanine aminotransferase	0	1 (4%)	0	0	1 (7%)	0
Increased creatinine	1 (3%)	0	0	0	0	0
Decreased neutrophil count	0	0	1 (13%)	0	0	0
**Metabolism and nutrition disorders**						
Anorexia	0	0	0	1 (6%)	0	0
Dehydration	0	0	0	1 (6%)	0	0
Hypernatraemia	0	0	0	1 (6%)	0	0
Hyponatraemia	0	0	1 (13%)	0	0	0
**Musculoskeletal and connective tissue disorders**						
Chest wall pain	0	0	0	0	1 (7%)	0
Myalgia	1 (3%)	0	0	0	0	0
Neck pain	1 (3%)	0	0	0	0	0
Extremity pain	1 (3%)	0	0	0	0	0
Tendonitis	3 (8%)	2 (8%)	0	0	0	0
Other	0	1 (4%)	0	0	0	0
**Nervous system disorders**						
Dizziness	1 (3%)	0	0	0	0	0
Peripheral sensory neuropathy	1 (3%)	0	0	0	0	0
Vasovagal reaction	0	1 (4%)	0	0	0	0
**Psychiatric disorders**						
Confusion	1 (3%)	0	0	0	0	0
Delirium	0	0	1 (13%)	0	0	0
**Renal and urinary disorders**						
Acute kidney injury	3 (8%)	0	1 (13%)	1 (6%)	0	1 (33%)
Chronic kidney disease	0	1 (4%)	0	0	0	0
**Respiratory, thoracic, and mediastinal disorders**						
Hypoxia	0	0	0	0	1 (7%)	0
**Skin and subcutaneous tissue disorders**						
Erythroderma	0	0	0	0	1 (7%)	0
Pruritus	1 (3%)	0	0	0	0	0
Maculopapular rash	2 (5%)	4 (15%)	0	1 (6%)	0	0
Skin ulceration	0	0	0	1 (6%)	0	0
Stevens-Johnson syndrome	0	0	0	0	0	1 (33%)
Other	0	2 (8%)	0	0	0	0
Missing	0	0	0	1 (6%)	0	0
**Vascular disorders**						
Hypotension	0	1 (4%)	0	0	0	0

Data are n (%; for each column denominator).

**Figure 2:**
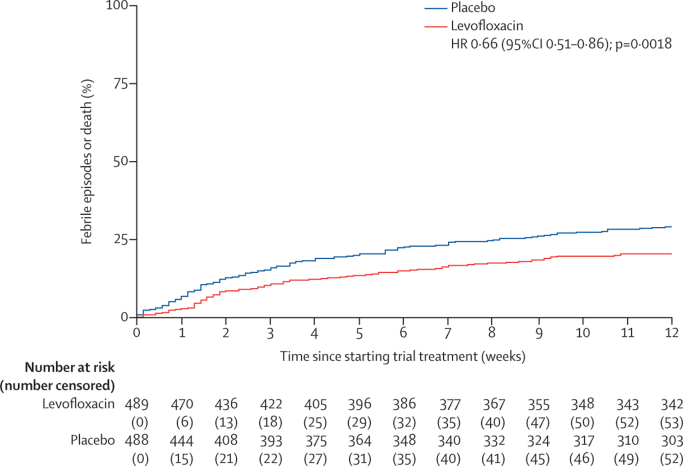
Kaplan-Meier graph of time to febrile episode or death

**Figure 3:**
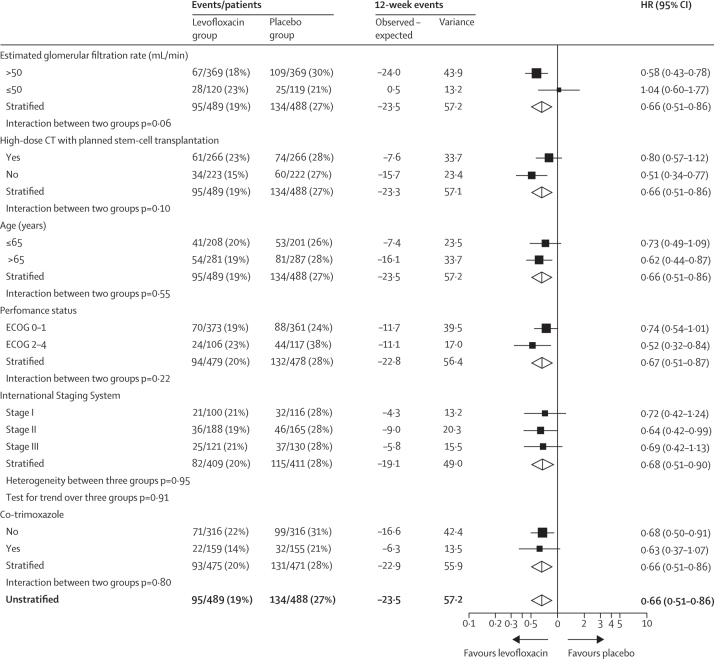
Forest plots of time to febrile episode or death in various subgroups ECOG=Eastern Cooperative Oncology Group. HR=hazard ratio.

In the levofloxacin group, 95 (19%) first febrile episodes or deaths in 489 patients were reported versus 134 (27%) in 488 patients in the placebo group (hazard ratio for time to first event within 12 weeks [HR] 0·66, 95% CI 0·51–0·86; p=0·0018; figure 2). Febrile episodes, deaths, and febrile episodes with death during the first 12 weeks were less frequent in the levofloxacin group than in the placebo group (87 [18%] *vs* 112 [23%] febrile episodes, four [1%] *vs* 15 [3%] deaths, and four [1%] *vs* seven [1%] febrile episodes with death; appendix p 6). A prespecified subgroup analysis of time to first febrile episode or death within the first 12 weeks of treatment is shown in figure 3.

Cox regression analysis showed that treatment with levofloxacin was the most important factor in reducing febrile episodes or deaths and retained significance even when controlling for baseline characteristics. 314 patients took co-trimoxazole 960 mg three times per week to prevent *Pneumocystis jirovecii* pneumonia (155 in the placebo group and 159 in the levofloxacin group; appendix p 10). We recorded only one case of proven *P jirovecii* pneumonia and one possible microbiologically unproven case (both in the levofloxacin group), and neither of these patients were receiving co-trimoxazole prophylaxis. Use of prophylactic low dose co-trimoxazole also showed a significant benefit in reducing febrile episodes and deaths (HR 0·59, 95% CI 0·44–0·80; p=0·0007) and adjustment for co-trimoxazole made little difference to the levofloxacin benefit (0·66, 0·51–0·86; p=0·0015), indicating that these two variables have independent effects (appendix p 10).

Median follow-up was 12 months (IQR 8–13). Log-rank analyses showed an overall survival benefit for the use of levofloxacin up to 12 weeks, with patients in the levofloxacin group having overall survival of 98% (95% CI 97–99, with 426 of 489 patients known to be surviving) versus overall survival in the placebo group of 95% (93–97, with 405 of 488 patients known to be surviving; p=0·0081), but no survival benefit at 12 months (levofloxacin overall survival 90%, 95% CI 87–93 *vs* placebo overall survival 91%, 88–93; p=0·94; appendix p 11). We observed no treatment-related deaths. An independent review of causes of death did not reveal differences between the two trial groups. During the 12-week trial period, two (25%) of eight patients in the levofloxacin group and ten (45%) of 22 in the placebo group died while known to be responding to treatment. Five (63%) of eight in the levofloxacin group and 14 (64%) of 22 patients in the placebo group died with no evidence of infection at the time of death. Deaths during the whole 12-month trial period were associated with progressive myeloma in 29 (73%) of 40 patients in the levofloxacin group and 15 (38%) of 39 patients in the placebo group, and infection in 15 (38%) of 40 patients in the levofloxacin group and 15 (38%) of 39 patients in the placebo group (table 3).

**Table 3:** Disease status and evidence of infection at time of death

		**Levofloxacin group**	**Placebo group**
**Deaths within 12 weeks**			
Disease status at death			
	Myeloma in remission or responding	2/8 (25%)	10/22 (45%)
	Myeloma active—progressing	2/8 (25%)	5/22 (23%)
	Myeloma active—unknown	4/8 (50%)	7/22 (32%)
Evidence of infection			
	No	5/8 (63%)	14/22 (64%)
	Yes	3/8 (37%)	8/22 (36%)
**Deaths between 12 weeks and 52 weeks**			
Disease status at death			
	Myeloma in remission or responding	5/32 (16%)	7/17 (41%)
	Myeloma active—progressing	27/32 (84%)	10/17 (59%)
Evidence of infection			
	No	20/32 (63%)	10/17 (59%)
	Yes	12/32 (37%)	7/17 (41%)
**Deaths between 0 weeks and 52 weeks**			
Disease status at death			
	Myeloma in remission or responding	7/40 (17%)	17/39 (44%)
	Myeloma active—progressing	29/40 (73%)	15/39 (38%)
	Myeloma active—unknown	4/40 (10%)	7/39 (18%)
Evidence of infection			
	No	25/40 (63%)	24/39 (62%)
	Yes	15/40 (37%)	15/39 (38%)

Data are n/N (%).

Measuring the total number of febrile episodes, 217 patients had a total of 264 febrile episodes and these showed benefit for the use of levofloxacin versus placebo with 92 (20%) of 471 patients affected in the levofloxacin group versus 125 (27%) of 463 affected in the placebo group (p=0·018). There were fewer hospital (88 *vs* 114) and intensive care unit admissions (three *vs* five) for infection in the levofloxacin group versus the placebo group. Sites of infection were predominantly reported in the respiratory tract (appendix p 7). At study entry, respiratory-related comorbidities including chronic obstructive pulmonary disease, asthma, and bronchiectasis were reported in 75 (15%) of 489 patients in the levofloxacin group and 67 (14%) of 488 patients in the placebo group.

There were 323 non-febrile infections requiring anti-infective treatment. These non-febrile infections occurred in 249 patients—116 (24%) of 489 patients in the levofloxacin group versus 133 (27%) of 488 patients in the placebo group (p=0·23). The sites of non-febrile infections were less frequent in the respiratory tract (94 [29%] of 323 lower respiratory tract and 43 [13%] of 323 upper respiratory tract) with more urinary tract (38 [13%] of 323) and skin and soft tissue infections (42 [13%] of 323) reported compared with febrile infections (data will be reported in full in a future publication.

112 organisms were isolated during the study (44 [39%] in the levofloxacin group *vs* 68 [61%] in the placebo group), comprising 77 bacterial, 20 viral, and 15 candida species samples (appendix p 8). Fewer Gram-negative organisms were isolated in the levofloxacin group than in the placebo group (appendix p 8). 59 (70%) of 84 organisms isolated from the levofloxicin group versus 124 (73%) of 170 organisms isoloated from the placebo group were sensitive to a range of antibiotics, although zero of three isolates in the levofloxacin group were sensitive to fluoroquinolones versus seven (88%) of eight patients in the placebo group (appendix p 9). A case of suspected pneumocystis pneumonia and a case of microbiologically proven pneumocystis pneumonia were reported in two (<1%) of 662 patients who had not taken co-trimoxazole in the levofloxacin group, and there was one case of *C difficile* infection in each of the two trial groups.

Most of the clinical isolates reported by local laboratories were bacterial, but 15 (13%) of 112 were *Candida* spp (mainly upper airway or oral infections) and 20 (18%) of 112 were viral (appendix p 8). There were fewer *Streptococcus pneumoniae* infections (usually levofloxacin-sensitive) than we anticipated, with three isolates reported in the placebo group and none in the levofloxacin group.

2595 stool and 2933 nasal samples were returned. We observed similar acquisition rates for carriage of *C difficile*, ESBL Gram-negative coliforms, and MRSA between the levofloxacin group and placebo group over the 12 weeks of treatment and 4 weeks after treatment (table 4). 25 acquisitions of carriage of these organisms were found in the levofloxacin group at baseline compared with 51 in the placebo group (table 4). This difference could not be accounted for by imbalance of baseline factors, by use of antibiotics in the month before diagnosis (75 [15%] of 489 patients in the levofloxacin group *vs* 76 [16%] of 488 patients in the placebo group), or by the use of steroids in the 14 days before trial entry (248 [51%] patients in the levofloxacin group *vs* 246 [50%] patients in the placebo group). Nevertheless, new acquisitions of health care-associated infections were similar between the trial groups (40 in the levofloxacin group *vs* 45 in the placebo group).

**Table 4:** Acquisition of carriage of *Clostridium difficile*, ESBL, and MRSA organisms

	**Levofloxacin group**			**Placebo group**		
	*C difficile*	ESBL	MRSA	*C difficile*	ESBL	MRSA
Present at baseline (785 stool, 928 nasal samples)	1	19	5	5	37	9
Week 4 (706 stool, 805 nasal samples)	4	8	0	3	11	4
Week 8 (662 stool, 759 nasal samples)	0	5	1	2	7	1
Week 12 (634 stool, 719 nasal samples)	3	3	1	2	7	2
Week 16 (593 stool, 650 nasal samples)	4	9	2	1	5	0
Total new acquisitions (2595 stool, 2933 nasal samples)	11	25	4	8	30	7

ESBL=extended-spectrum β-lactamase. MRSA=methicillin-resistant *Staphylococcus aureus*.

## Discussion

Levofloxacin prophylaxis resulted in significantly fewer deaths, febrile episodes, and a longer time to first febrile episode or death, than did treatment with placebo. This difference remained significant after adjustment for baseline prognostic factors, including the use of prophylactic co-trimoxazole and when stratifying by various subgroups.

Conventionally, studies on antibiotic prophylaxis in neutropenia use febrile episodes or infected patients as the primary endpoint, because studies are not usually powered to show a predicted 2–3% reduction in mortality.^10^ Nevertheless, death is an important endpoint and should be captured. If febrile episodes alone is the primary endpoint, a higher number of deaths in one randomisation group might favour this group, since death prevents further febrile episodes occurring in this group. Our combined primary endpoint overcame this problem.

The TEAMM trial showed a significant reduction in deaths with the use of levofloxacin compared with placebo within 12 weeks. To our knowledge, no single large well-conducted study on antibiotic prophylaxis in neutropenia has shown a significant reduction in deaths, but meta-analysis showed a 3% reduction with fluoroquinolones and is the basis of several guidelines in patients with neutropenia.^10^, ^15^, ^17^ The reduction in deaths observed in the TEAMM study suggests that either the benefit of levofloxacin prophylaxis in newly diagnosed myeloma might be greater than in prolonged neutropenia, which could be shown in a future meta-analysis, or that our trial sample was just sufficient to show a benefit statistically and the real size of benefit might be similar to that in prolonged neutropenia. Either way, to our knowledge this is the first time that the use of prophylactic antibiotics has shown a survival benefit in patients with newly diagnosed myeloma.

The reduction in early deaths began within the first month and continued throughout the 3-month treatment period, during which there were 30 deaths. A further 49 deaths occurred by 12 months, by which time there was no survival benefit for the levofloxacin group. There may be additional mechanisms underlying the benefits of levofloxacin prophylaxis other than a reduction in infection. A change in the microbiome might have contributed to a reduction in inflammation. In-depth studies on the microbiome were not part of this trial but are planned for the future. Levofloxacin might have kept non-responding patients alive longer than placebo patients and once the treatment stopped non-responders began to die. If this were the case, continuing levofloxacin beyond 12 weeks might keep a patient who did not respond to first-line therapy alive long enough to respond to second-line therapy.

A 12-week prophylaxis period was chosen from data available at trial initiation. These data showed that the infection risk was highest in the first 2 months after diagnosis with myeloma and reduced as the disease responded to treatment and came under control.^7^ These data were from patients treated before the use of novel anti-myeloma agents. More recent studies have suggested that novel agents and high-dose steroids might contribute to a persistent risk of infection, even when myeloma is well controlled and this infection risk remains high during the first year after diagnosis.^6^ With the introduction of immunotherapy, induction regimens might become even more immunosuppressive and be continued for longer periods than at present, increasing the risk of infection-related death. A continuing infection risk beyond 12 weeks raises the question of whether the absence of survival benefit at 12 months might be due to early stopping of the intervention—12 months of prophylaxis might be beneficial.

A limitation of our study is that the total number of deaths was small, reflecting a generally well and young patient population. 2011–15 population data for England showed 79·2% net survival for newly diagnosed patients with myeloma at 12 months.^9^ 12-month overall survival in our study was 90%, and if adjusted for the risk of unrelated death as with net survival, the difference between our trial population and real-world population survival would be greater. Since our subgroup analysis suggested that the benefit of levofloxacin was greatest in older and less fit patients, levofloxacin prophylaxis might exert a greater benefit once it is adopted in a real-world population. Furthermore, since a benefit for prophylaxis was shown in a typical trial population that comprises a younger, more favourable myeloma prognosis group than does real-world patients, there is no patient subgroup for which levofloxacin prophylaxis should not be recommended. However, some subgroup investigations might have been underpowered. Although a 3% reduction in mortality at 12 weeks with levofloxacin prophylaxis might seem small, this translates into 165 deaths prevented in the UK per year. Recommendation of levofloxacin prophylaxis should be considered in the context of the incidence of local levofloxacin resistance in other countries. In the UK in 2017, the prevalence of *Escherichia coli* resistance to fluoroquinolones was reported to be 17·5%, but in Italy resistance is 47%.^25^ However, such high resistance might not be observed for all relevant invasive organisms in myeloma. Prophylactic levofloxacin efficacy is likely to be lower in countries with high levels of antibiotic resistance. In such countries, patient subgroups showing the strongest benefit for levofloxacin prophylaxis, namely patients who are not eligible for stem cell transplantation and those with Eastern Cooperative Oncology Group performance status 2–4, are still likely to benefit.

Febrile episodes with a temperature of 38°C or greater are not a trivial event for patients with myeloma and have a major impact on quality of life and health-care costs, as patients are requested to attend hospital and are frequently admitted. We also collected data on all non-febrile infections. To our knowledge, data on non-febrile infections have not been collected before on such a large scale. We observed more non-febrile infections than febrile infections (323 non-febrile *vs* 264 febrile), showing such infections also have a substantial burden for patients and the health-care system. The clinically attributed sites of infection during febrile episodes were similarly distributed to those previously reported in the literature,^26^, ^27^ with most being in the respiratory tract.

Levofloxacin was well tolerated, and related serious adverse events were rare. European Medicines Agency^17^ and US Food and Drug Administration alerts^28^ have highlighted the risk of long-term tendon damage, neuropsychiatric concerns, and hypoglycaemia following treatment with fluoroquinolones, especially in elderly people and largely occurring when the cause of such symptoms was not recognised and drug treatment continued. Our data showed a less than 1% risk of tendonitis with no or mild sequelae after stopping levofloxacin, despite our median patient age being 67 years and most patients receiving high-dose steroids. Close clinical supervision and regular reassessment of estimated glomerular filtration rate and appropriate levofloxacin dose might have been important in preventing substantial toxic effects. The benefits of levofloxacin outweighed the minimal toxic effects in this patient population. Patients who received 250 mg levofloxacin because of an estimated glomerular filtration rate of 50 mL/min or less did not derive a benefit of levofloxacin prophylaxis in our subgroup analyses. Although some hospitals might use levofloxacin 250 mg daily for patients with neutropenia, there is no evidence that this dose is sufficient and therefore we recommend the use of adjusted therapeutic doses of levofloxacin as per estimated glomerular filtration rate according to the manufacturer's instructions.

314 patients were given co-trimoxazole 960 mg three times per week to prevent *P jirovecii* infection. There was only one confirmed and one microbiologically unproven case of *P jirovecii* infection. A previous antibiotic prophylaxis study in patients with myeloma also reported no *P jirovecii* pneumonia in 212 patients, although two-thirds of patients were not receiving co-trimoxazole.^19^ Therefore, we conclude that the risk of *P jirovecii* pneumonia in patients with newly diagnosed myeloma is low and probably does not warrant routine treatment with prophylactic co-trimoxazole. The finding that unrandomised use of low-dose co-trimoxazole might have an independent beneficial effect on febrile episodes and death is intriguing. Even at a low dose, co-trimoxazole might have a prophylactic antibacterial effect and could have contributed to the low incidence of *S pneumoniae* infection. Since co-trimoxazole use was unrandomised and determined by the treating centre, these findings therefore could be spurious and should be interpreted with caution. However, the addition of a complementary antibiotic could be beneficial and should be investigated in future studies.

The difference between our study and previous studies in patients with myeloma that reported no benefit for antibiotic prophylaxis might be due to the greater number of participants in the TEAMM trial and differences in the anti-myeloma treatments used. To our knowledge, the largest study to date took 10 years to recruit 212 patients and most of these patients did not receive novel anti-myeloma agents, although some received high-dose dexamethasone.^19^ Our study recruited participants rapidly with broad entry criteria that allowed all forms of anti-myeloma treatment—98% of our patient population received novel agents, which therefore reflects current practice. The use of total febrile episodes as a primary endpoint might also have contributed to a negative study, as previously discussed.

The viral isolates reported in our study are probably an under-representation of viral infections, since taking specimens for viral identification was not routine practice in many district hospitals during the trial period. However, the protocol stipulated taking local microbiological specimens. The benefit of levofloxacin in reducing bacterial infections appears to be largely associated with Gram-negative rather than Gram-positive organisms. The small number of *S pneumoniae* infections might have partly resulted from 155 placebo patients taking co-trimoxazole 960 mg three times per week to prevent *P jirovecii* pneumonia. Furthermore, there has been an increase in pneumococcal vaccination in the UK, with the 23-valent polysaccharide pneumococcal vaccine offered at 65 years of age since 2003 and herd immunity boosted by conjugate pneumococcal vaccines for children younger than 5 years with a seven-valent vaccine introduced in 2006 and 13-valent vaccine introduced in 2010.

The perceived risks of increased carriage of antibiotic-resistant organisms and health care-associated infections were not evident in our study results—we observed no increase in colonisation with antibiotic-resistant organisms or health care-associated infections in the levofloxacin groups, suggesting that there might be situations when the use of prophylactic antibiotics does not increase the carriage of antibiotic-resistant organisms. This observation is supported by large reviews and meta-analyses of prospective studies on antibiotic prophylaxis^10^, ^29^ or selective digestive decontamination,^30^, ^31^, ^32^ which did not show increased carriage of resistant isolates compared with placebo groups. Our study group differs from previous studies because most patients were not admitted to hospital in our study.

The main limitation of this study is the high number of withdrawals, despite the low toxicity. Around half of the withdrawals occurred in the first 4 weeks, and the high numbers might reflect poor commitment of patients with newly diagnosed myeloma to a supportive care placebo-controlled trial, possibly because they prioritise focusing on disease control. Patients had to collect stool samples every 4 weeks, take their temperatures daily, and fill in a diary, which might have been burdensome. Myeloma XI^33^ was an open-label randomised trial of anti-myeloma therapies that ran concurrently with TEAMM, recruited at three times the rate of TEAMM, and had fewer withdrawals. 40% of the patients in the TEAMM trial were also enrolled in Myeloma XI and prioritised compliance with active anti-myeloma therapy rather than placebo-controlled infection prophylaxis. A previous supportive care trial in patients with myeloma who received oral clodronic acid or placebo had higher withdrawal than the TEAMM trial (209 [39%] of 536 patients).^34^ High withdrawal could be a feature of placebo-controlled supportive care trials and might require consideration in future recruitment targets. However, once an intervention is observed to be effective in reducing deaths, as levofloxacin was in this trial, compliance might be expected to be good. Suspected drug toxic effects accounted for a quarter of withdrawals in the TEAMM trial and were balanced between the study groups, suggesting that perceived rather than actual toxic effects might have contributed to the large number of withdrawals.

In summary, our study found a consistent benefit for the use of levofloxacin prophylaxis over 12 weeks in patients with newly diagnosed myeloma, without any increased in health care-associated infections. Thus, patients with newly diagnosed myeloma could benefit from levofloxacin prophylaxis, although local antibiotic resistance proportions must be considered. Prolonged antibiotic prophylaxis after 12 weeks and combined antibiotic use for prophylaxis requires investigation in future studies.

## Data sharing

Participant data is stored on a secure server at Warwick Clinical Trials Unit (Coventry, UK) where each participant has been assigned a deidentified trial number. No identifiable data, such as name, address, hospital number, NHS number, date of birth, or any other identifying data, will be shared and should not be requested. A data dictionary will be available and will include descriptions of patient demographics, treatment allocation, and primary outcome data. Any requests for access to the TEAMM trial data should be sent to the chief investigator (m.t.drayson@bham.ac.uk) who will inform the data custodians (ie, Warwick Clinical Trials Unit) and agreement will be made through the data access committee, which will comprise the principal investigators from the trial management group. For each data sharing request, it is essential that a proforma is completed that describes the purpose, scope, data items requested, analysis plan, and acknowledgment of the trial management team. Requestors who are granted access to the data will be required to complete a data sharing agreement that will be signed by the requester, sponsor, and principal investigator(s). The study protocol, statistical analysis plan, and consent forms are available upon request.
