# Supplementary material for: Levofloxacin prophylaxis in patients with newly diagnosed myeloma (TEAMM): a multicentre, double-blind, placebo-controlled, randomised, phase 3 trial
Source: Lancet Oncol. 2019 Dec;20(12):1760–72. doi: 10.1016/S1470-2045(19)30506-6 (PMC6891230; doi:10.1016/S1470-2045(19)30506-6)
Supplement: Supplementary appendix [file mmc1.pdf]

THE LANCET
Oncology

Supplementary appendix

This appendix formed part of the original submission and has been peer reviewed. We post it as supplied by the authors.

Supplement to: Drayson M T, Bowcock S, Planche T, et al. Levofloxacin prophylaxis in patients with newly diagnosed myeloma (TEAMM): a multicentre, double-blind, placebo-controlled, randomised, phase 3 trial. *Lancet Oncol* 2019; published online October 23. https://doi.org/10.1016/S1470-2045(19)30506-6.

Table S1: List of TEAMM trial investigators by site in order of recruitment

Site	Investigators	No Randomised
Queen Alexandra Hospital	Helen Dignum, Alison Cook, Mary Ganczakowski, Kavitha Sooriyekumen, Charles Alderman, Katie Smith, Robert Corser, Edward Belsham, Gwynn Matthias, Inga Slepikiene, Izabela James, James Croft, Tanya Cranfield, Christopher James	51
Royal Stoke University Hospital	Kamaraj Karunanithi, Neil Phillips	39
Russells Hall Hospital	Stephen Jenkins, Rupert Hipkins, Savio Fernandes, Craig Taylor, Jeff Neilson	38
University Hospital, Coventry	Anand Lokare, Syed Bokhari, Beth Harrison, Nick Jackson, David Lewis, Oliver Chapman, Rabia Afghan	32
Colchester General Hospital	Michael Hamblin, Gavin Campbell, Marion Wood, Sudhakaran Makkuni, Mahalakshmi Mohan Pavel Kotouchek	31
Princess Royal University Hospital	Stella Bowcock, Anil Lakhani, Corrine DeLord, Mansour Ceesay, Siamak Arami, K Yip	31
St Helier Hospital	Simon Stern, Lydia Jones, Sneha Muthalali, Nicholas Fordham, Jane Mercieca, Shalini Solanki, Charlotte Pawlyn, Priya Sriskandarajah, Sandra Easdale, Ying Ying Peng	28
Medway Maritime Hospital	Maadh Aldouri, Sarah Arnott, Handunneththi Mendis, Vivienne Andrews, Waseem Nagi, Ayed Eden, Vijayavalli Dhanapal	26
Kings Mill Hospital	Rowena Faulkner, Ashish Masurekar, Tim Moorby	25
Sandwell General Hospital	Farooq Wandroo, Richard Murrin, Shivan Pancham, John Gillson, Yasmin Hasan	25
Darent Valley Hospital	Anil Kamat, Tariq Shafi, Raphael Ezekwesili, Pavel Kotoucek, Lalita Banerjee	23
Heartlands Hospital	Guy Pratt, Bhuvan Kishore, Don Milligan, Joanne Ewing, Clare Gardner, Hauder Hussein, Jonathan Lancashire, Manos Nikolousis, Neil Smith, Richard Lovell, Shanhara Paneesha	21
Northampton General Hospital	Alistair McGrann, Angela Bowen, Jane Parker, Sajjan Mittal, Suchi Krishnanurthy, George Cherian, Philip George, Rebecca Allchin	19
Ulster Hospital, Belfast	Jeremy Hamilton, Moulod El-Agnaf, Yong Lee Ong, Adnan Alshoufi, Anna Elliott, Barbara Black, Margaret Bowers	18
Wrexham Maelor Hospital	Lally Desoysa, David Watson	18
Castle Hill Hospital	David Allsup, Haz Sayala, Senthil Durairaj, Andrew Fletcher, Safia Dawi	17
Guy's and St Thomas' Hospital	Matthew Streetly	17
Salisbury District Hospital	Jonathan Cullis, Louise Fraser, Tamara Everington, Effie Grand, Freya Collings	17
West Middlesex University Hospital	Magda Jabbar Al-Obaidi,	17

	Anna Babb, Andrew Godfrey, Andrew Hastings, John Riches	
Queen Elizabeth Hospital, Kings Lynn	Lisa Cooke, Ramaprabhahari Satheshkumar, Emma Gudgin, Kway Zin Maw, Martin Lewis, Joseph Padayatty, Mark Robinson	16
Southampton General Hospital	Matthew Jenner, Srinivasan Narayanan	14
Southmead Hospital	Alastair Whiteway, Samreen Siddiq, Miloslav Kmonicek	14
Chesterfield Royal Hospital	Peter Toth, Andrew Fletcher, Emma Welch, Mark Wodzinski, Sandor Lueff	13
Great Western Hospital	Norbert Blesing, Hala Ahmed, Alex Sternberg	13
Queen Elizabeth Hospital, Birmingham	Yu Sandar Aung, Mark Cook, Timothy Wong, David Burns, Manoj Raghavan	13
Queen's Hospital, Romford	Biju Krishnan, Claire Hemmaway, Paul Greaves	13
Royal Berkshire Hospital	Sadon Hassan, Henri Grech, Karthik Ramasamy, Michael Desborough, Rebecca Sampson	13
Stoke Mandeville Hospital	Helen Eagleton, Anne-Marie O'Hea, Beena Pushkaran, Ann Watson, Naima Ansari	13
Ealing Hospital	Mamta Sohal, Richard Kaczmarski, Christine Liu, Angharad (Hari) Pryce, Janeta Grigorova	12
Manchester Royal Infirmary	Alberto Rocci, Simon Gibbs, Eleni Tholouli, Rajesh Krishna	12
Royal United Hospital, Bath	Josephine Crowe, Sally Moore, Amy Knott, Jane Norman, Jennifer Page, Karan Wadehra	12
Altnagelvin Hospital	Feargal McNicholl, Patrick Elder, Curly Morris	11
Lincoln County Hospital	Kandeepan Saravanamuttu, Charlotte Kallmeyer, Caroline Harvey	11
Northwick Park Hospital	Charalampia Kyriakou, Robert Ayto, Nicki Panoskaltsis	11
Southend University Hospital	Paul Cervi, Mohammed Islam	11
St James University Hospital	Gordon Cook, Charlotte Kallmeyer, Christopher Parrish	11
Wythenshawe Hospital	Sumaya Elhanash, Shiva Kumar Natarajan, Simon Watt	11
Stafford Hospital (Stafford)	Paul Revell, Avrangzeb Razzak, Andrew Amos, Ghulam Kakepoto, Mohammed Hameed	10
Glangwili General Hospital	Sonya Goriah, Peter Cumber, Praba Gupta	10
North Middlesex University Hospital	Neil Rabin	10
Royal Devon and Exeter Hospital	Claudius Rudin, Tony Todd, Loretta Ngu, Paul Kerr	10
Wycombe Hospital	Beena Pushkaran, Robin Aitchson	10
Calderdale Royal and Huddersfield Royal Infirmary	Sylvia Feyler, Nwe Oo, Wunna Swe, Sylvia Feyler,	9
New Cross Hospital	Supratik Basu, Sophie Lee	9
Bradford Royal Infirmary	Adrian Williams, Anshu Garg, Nandini Sadasivam, William Wong	8
Broomfield Hospital	Vijoy Chowdhury, Finella Brito, Shereen Elshazly, Waseem Nagi	8

Warrington Hospital	Chandramouli Nagarajan, Jeyaprakash Ramachandran, Mohamed Kaleel Rahman	8
Hereford County Hospital	Christopher Branner, Lisa Robinson, Sara Willoughby	7
Leicester Royal Infirmary	Claire Chapman, Mamta Garg	7
Milton Keynes Hospital	Moez Dungaewalla, Subir Mitra	7
Pilgrim Hospital Boston	Ciro Rinaldi, Juan Contesti, Mohammed Hameed	7
Royal Hampshire County Hospital	Jennifer Arnold, Katherine Lowndes, Nigel Sargant	7
Royal Surrey County Hospital	Johannes De Vos, Elizabeth Grey-Davies, Louise Hendry	7
Craigavon Area Hospital	Kathryn Boyd, Donald Hull, Frank Jones, Hakim Eswedi, Jonathan Palmer	6
George Eliot Hospital	Mekkali Narayanan, Jagadeesh Gandla, Jhansi Muddana	6
Good Hope Hospital	Vidhya Murthy, Bhuvan Kishore, Richard Lovell	6
Queens Hospital, Burton	Jawaid Channa, Humayun Ahmad, Bernhard Van Staden	6
Royal Hallamshire Hospital	Andrew Chantry	6
Sunderland Royal Hospital	Shikha Chattree, Susanna Matthew, Victoria Hervey	6
Basildon University Hospital	Joanne Howard, Parag Jasani	5
Gloucestershire Royal Hospital	Michael Sheilds, Asha Johny, Phil Robson, Sally Chown	5
University Hospital, Lewisham	Naheed Mir, Tullie Yeghen, Murugaiyan Thanigaikumar	5
Warwick Hospital	Carolina Arbuthnot, Katie Randall, Anton Borg	5
Wexham Park Hospital	Mark Offer, Carolina Lahoz, Nicola Bienz	5
Antrim Hospital	Philip Windrum, Joanne Murdock, Scott McCloskey	4
King's College Hospital	Steve Schey, Guillermo Orti	4
Kingston Hospital	Sangeetha Atwal, Samir Zebari, Vishal Jayakar	4
Royal Liverpool University Hospital	Nauman Butt, Stephen Hawkins	4
Torbay Hospital	David Tucker, Deborah Turner, Steve Smith	4
Whipps Cross Hospital	Sajida Kazi, Syed Rabbani	4
Basingstoke and North Hampshire Hospital	Noel Ryman	3
Frenchay Hospital	Samreen Siddiq, Alastair Whiteway	3
Leighton Hospital	Kamaraj Karunanithi	3
Queen Elizabeth Hospital (London)	Ana Duran, Betty Cheung	3
Royal Bournemouth Hospital	Rachel Hall	3
Withybush Hospital	Sumant Kundu	3
Dewsbury & District Hospital	Mary Chapple	2
Diana, Princess of Wales Hospital	Susan Levison-Keating	2
Dorset County Hospital	Akeel Moosa	2
Grantham & District Hospital	Caroline Harvey	2

Hillingdon Hospital	Richard Kaczmarski	2
Kettering General Hospital	Mark Kwan, Matthew Lyttelton	2
Pinderfields General Hospital (Wakefield)	Paul Moreton, Praveen Kaudlay	2
Poole Hospital	Fergus Jack	2
Pontefract General Infirmary	David Wright	2
Royal Shrewsbury Hospital	Emma Litt, Stephen McKew	2
Glan Clwd Hospital	Earnest Heartin	1
Macclesfield District General Hospital	John Hudson	1
Royal Gwent Hospital	Jessica Anderson	1
North Bristol Hospital	-	0
North Devon District Hospital	-	0
Tameside General Hospital	-	0
**Grand Total**		**977**

Table S2: SAE severity assessment by treatment arm

Severity	Levofloxacin (N=308)<br><br>N (%)	Placebo(N=289)<br><br>N (%)	Total (N=597)<br><br>N (%)
Mild	38 (12)	29 (10)	67 (11)
Moderate	136 (44)	141 (49)	277 (46)
Severe	101 (33)	86 (30)	187 (31)
Fatal/life-threatening	32 (10)	33 (11)	65 (11)
Unknown	1 (<1)	0 (0)	1 (<1)

Table S3: Combined events (first febrile episodes and deaths) by treatment arm

Event category	Levofloxacin (n=489) N	Placebo (n=488) N	Total (n=977) N
None	394	354	748
Febrile episode only	87	112	199
Death only	4	15	19
Febrile episode and death	4	7	11
**Total events N (%)**	**95 (19%)**	**134 (27%)**	**229 (23%)**

Table S4: Sites of febrile infection by treatment arm

	Levofloxacin		Placebo		Total	
**Site of infection**	**N**	**%**	**N**	**%**	**N**	**%**
Lower respiratory tract	39	47	49	51	88	49
Upper respiratory tract	11	13	11	11	22	12
Intravenous catheter associated	1	1	1	1	2	1
Bloodstream	5	6	8	8	13	7
Urinary tract infection	6	7	7	7	13	7
Gastrointestinal tract	2	2	3	3	5	3
Skin soft tissue	6	7	8	8	14	8
Other site	7	8	7	7	14	8
Unknown	6	7	1	1	7	4

There were 14 'other' reported sites of febrile infections (7 levofloxacin and 7 placebo). The 7 reported on levofloxacin being: Suspected Meningitis, Acute kidney infection, Diverticular abscess, Generalised myalgia possibly viral, Hospital Acquired Pneumonia-AKI both on diagnosis, Reaction to either Allopurinol or thalidomide, Intraoperative tear during spinal surgery where patient spiked temp after surgery, infection suspected. The 7 reported on the Placebo were: Neutropenic sepsis (2 patients), Neutropenic sepsis + Anaphylaxis type reaction, Neutropenic sepsis allergic reaction to medication, Acute cholecystitis, Fever and Atrial fibrillation, Testicles.

Table S5: Reported potentially pathogenic or invasive isolates from local laboratories by treatment arm

Species	Levofloxacin	Placebo	Total
**Total Gram negative**	**6 (18%)**	**27 (82%)**	**33**
Enterobacteriaciae	4	14	18
Pseudomonas Spp.	0	5	5
Other Gram negative	2	8	10
**Total Gram positive**	**16 (44%)**	**20 (56%)**	**36**
Staphylococcus aureus	4	6	10
Streptococcus pneumoniae	0	3	3
Coagulase negative staphylococus	5	5	10
Other Gram positive	7	6	13
**Total other bacterial**	**4 (50%)**	**4 (50%)**	**8**
Anaerobe	1	2	3
Mixed growth	3	2	5
**Total Viral**	**10 (50%)**	**10 (50%)**	**20**
**Adenovirus**	**1**	**0**	1
**Cytomegalovirus**	**1**	**0**	1
Herpes simplex/Varicella	3	0	3
Influenza	2	5	7
Metapneumovirus	0	2	2
Parainfluenza	2	3	5
Respiratory Syncytial Virus (RSV)	1	0	1
Candida* species	**8 (53%)**	**7 (47%)**	**15**
**Total isolates**	**44 (39%)**	**68 (61%)**	**112**

*Note: majority (14/15) oral/upper respiratory samples

Table S6: Antibiograms by treatment arm

	Levofloxacin		Placebo	
**Antibiotic category**	**Number sensitive /number tested**	**%**	**Number sensitive /number tested**	**%**
Quinolone	0/3	0	7/8	88
Penicillin	10/19	53	16/30	53
Aminoglycoside	5/7	71	11/13	85
Co-amoxiclav	4/5	80	9/14	64
Piperacillin Tazobactam	2/2	100	7/8	88
Carbapenem	2/2	100	6/6	100
Cephalosporin	2/2	100	6/8	75
Other	34/44	76	62/83	75
Total	59/84	70%	124/170	73%

Figure S1: Treatment arm stratified by Co-trimoxazole

a) Co-trimoxazole

b) No Co-trimoxazole

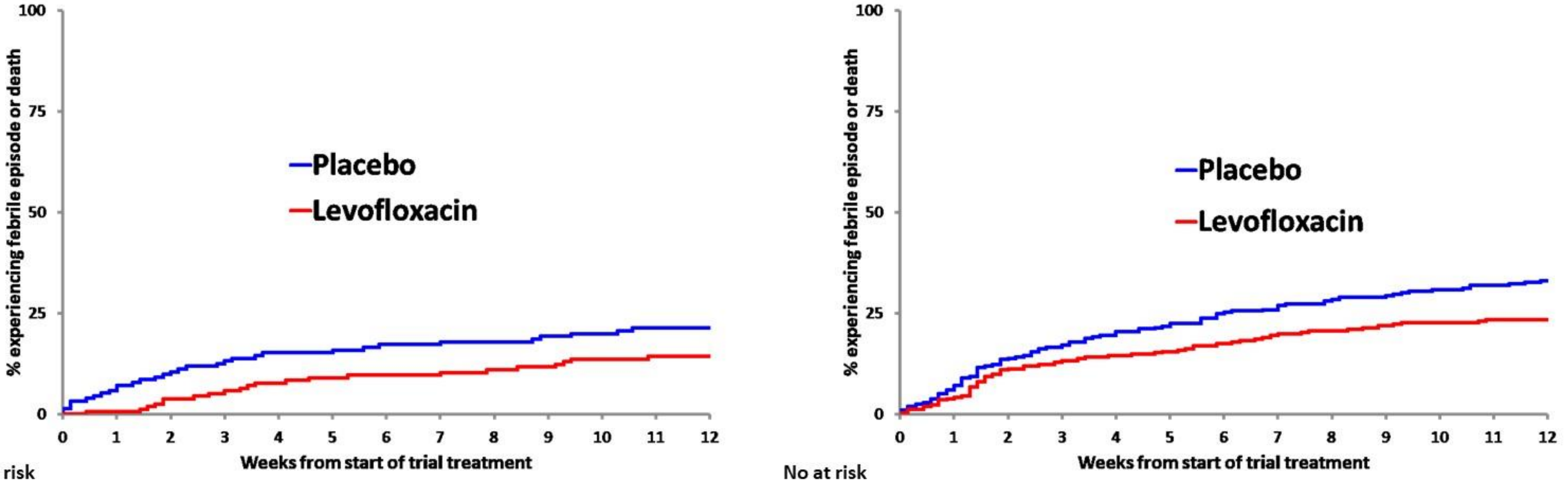

a) Co-trimoxazole — No at risk

Placebo	155	143	136	132	127	126	122	120	119	117	116	112	112
Levofloxacin	159	158	151	146	141	139	137	136	134	131	128	127	126

b) No Co-trimoxazole — No at risk

Placebo	316	291	264	254	241	231	219	213	206	199	193	190	185
Levofloxacin	316	301	274	268	259	252	244	236	228	220	215	211	211

Figure S2: Overall survival up to 12 months

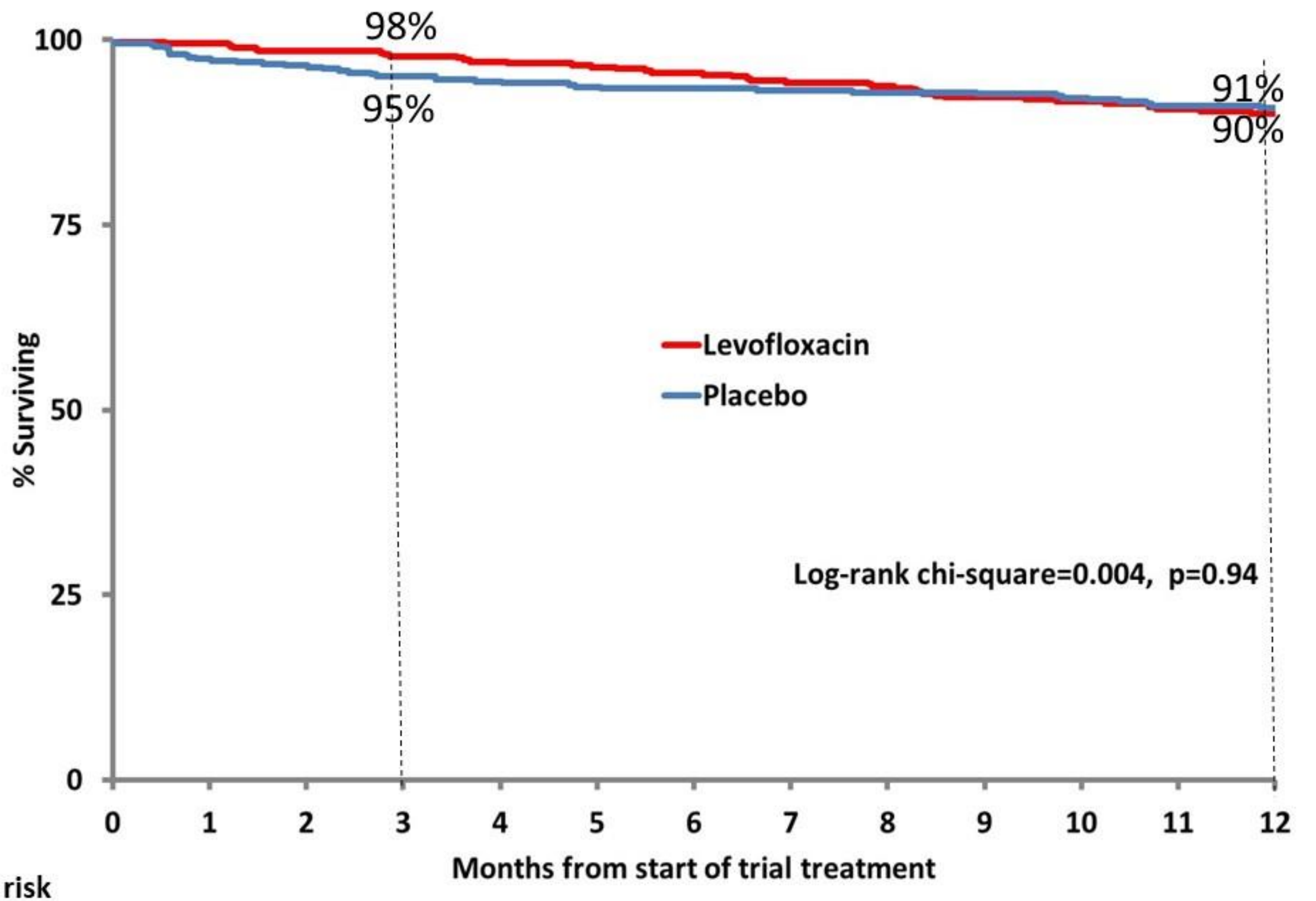

No at risk

Levofloxacin	489	461	439	426	401	380	374	371	368	363	359	356	342
Placebo	488	446	420	405	393	378	373	373	371	370	369	365	356
